# Safety and effectiveness of pirfenidone combined with carboplatin‐based chemotherapy in patients with idiopathic pulmonary fibrosis and non‐small cell lung cancer: A retrospective cohort study

**DOI:** 10.1111/1759-7714.13675

**Published:** 2020-09-28

**Authors:** Yuji Yamamoto, Yukihiro Yano, Tomoki Kuge, Fukuko Okabe, Mikako Ishijima, Takeshi Uenami, Masaki Kanazu, Yuki Akazawa, Toshihiko Yamaguchi, Masahide Mori

**Affiliations:** ^1^ Department of Thoracic Oncology National Hospital Organization Osaka Toneyama Medical Center Toyonaka Japan

**Keywords:** Acute exacerbation, immune checkpoint inhibitors, interstitial pneumonia, platinum‐based chemotherapy, toxicity

## Abstract

**Background:**

Pirfenidone is an antifibrotic agent that is potentially effective for the treatment of idiopathic pulmonary fibrosis (IPF). However, no study has reported on its prophylactic value against chemotherapy‐associated acute IPF exacerbations when combined with chemotherapy for non‐small cell lung cancer (NSCLC). The present study assessed the safety and effectiveness of pirfenidone combined with carboplatin‐based chemotherapy or immune checkpoint inhibitors (ICIs) in patients with IPF and NSCLC.

**Methods:**

A total of 14 patients with IPF and NSCLC who received treatment from 2013 to 2019 were included. Patients were treated with pirfenidone combined with carboplatin and nanoparticle albumin‐bound paclitaxel or S‐1 as first‐line chemotherapy. After confirming disease progression, patients received cytotoxic agents or ICIs, including nivolumab and pembrolizumab. Pirfenidone was continued regardless of chemotherapy changes. Overall survival (OS) and progression‐free survival (PFS) for lung cancer and IPF were calculated. Moreover, the cumulative incidence of acute exacerbation of IPF (AE‐IPF) within one year was evaluated.

**Results:**

Median PFS for lung cancer was 110 days (95% confidence interval [CI]: 57–199 days), while the median OS was 362 days (95% CI: 220–526 days). Moreover, PFS for IPF was 447 days (95% CI: 286–indeterminate days), and the cumulative incidence of AE‐IPF within one year was 18%. Notably, none of the patients developed AE‐IPF associated with first‐line chemotherapy. Among the included patients, four received ICIs, none of whom developed ICI‐associated AE‐IPF.

**Conclusions:**

The present study found that pirfenidone combined with carboplatin‐based regimens or ICIs might be safe first‐line chemotherapy for patients with IPF and NSCLC.

**Key points:**

**Significant findings of the study:**

- No patients with IPF and NSCLC who received pirfenidone in combination with first‐line carboplatin‐based chemotherapy or late‐line ICIs developed acute IPF exacerbations.

**What this study adds**

- Pirfenidone might have a prophylactic effect against chemotherapy‐associated AE‐IPF.

## Graphical abstract

The safety and effectiveness of pirfenidone combined with cytotoxic agents or immune checkpoint inhibitors (ICIs) in patients with idiopathic pulmonary fibrosis (IPF) and non‐small cell lung cancer (NSCLC) have not been investigated. This study showed that pirfenidone combined with carboplatin‐based chemotherapy or ICIs was safe for patients with IPF and NSCLC and did not induce acute exacerbations of IPF.

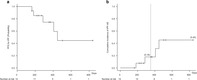

## Introduction

Idiopathic pulmonary fibrosis (IPF), also known as cryptogenic fibrosing alveolitis, is a specific form of chronic, progressive, fibrosing interstitial pneumonia of unknown cause with a poor prognosis.^1^ Since the clinical course of IPF varies, many studies on prognostication, including the gender, age, and physiology (GAP) index, have been reported.^2^, ^3^, ^4^, ^5^, ^6^ Moreover, antifibrotic agents, including pirfenidone and nintedanib, have been developed to delay the progression of IPF.^7^, ^8^, ^9^ These antifibrotic agents have been reported to inhibit the decline in forced vital capacity (FVC),^7^, ^8^ a common surrogate marker of overall survival (OS) in patients with IPF.^5^ As other benefits of antifibrotic agents, both pirfenidone and nintedanib prevent acute exacerbation of IPF (AE‐IPF).^10^, ^11^

Apart from progressive fibrosis, a high incidence of lung cancer in patients with IPF has also been reported, which is almost five times higher than that of healthy subjects.^12^, ^13^ Notably, patients with combined pulmonary fibrosis and emphysema (CPFE) and lung cancer showed poorer prognosis than those with IPF and lung cancer.^14^ Similar to healthy subjects, patients with IPF typically develop squamous cell carcinoma and adenocarcinoma,^15^ with lung cancer promoting further deterioration in their prognosis.^13^ Hence, earlier studies investigated the utility of chemotherapy for patients with IPF and non‐small cell lung cancer (NSCLC).^16^, ^17^ Some studies found that certain chemotherapy regimens using carboplatin were feasible, despite occasionally inducing AE‐IPF.^6^, ^16^, ^17^, ^18^, ^19^, ^20^ Even treatment regimens with less risk for AE‐IPF, including carboplatin, S‐1, paclitaxel, and vinorelbine, induced AE‐IPF at a rate of 10%–30%.^16^, ^18^, ^19^, ^20^ Therefore, a modified GAP (mGAP) index has been developed to predict AE‐IPF in patients with IPF and NSCLC.^6^ Although patients classified as mGAP stage I showed better prognosis and lower AE‐IPF incidence than those classified as mGAP stage II,^6^ they had a median OS and a one‐year cumulative AE‐IPF incidence of 10.3 months and 30%, respectively,^6^ which were still poorer than those for patients who had IPF without lung cancer.^13^ Thus, assessing novel applications for antifibrotic agents could be important in patients with IPF and NSCLC.

Given that antifibrotic agents prevent AE‐IPF, combining them with cytotoxic agents and/or immune checkpoint inhibitors (ICIs) might be safe and effective for patients with IPF and NSCLC. In fact, a phase I trial reported that chemotherapy with nintedanib, carboplatin, and nanoparticle albumin‐bound (nab)‐paclitaxel was safe to administer to patients with NSCLC.^21^ Moreover, a novel randomized controlled trial for combined chemotherapy with nintedanib, carboplatin, and nab‐paclitaxel for patients with IPF and NSCLC has been launched.^22^ With regard to pirfenidone, it has been reported to prevent perioperative AE‐IPF in patients with IPF and lung cancer;^23^ however, no study has previously reported on its prophylactic value when combined with chemotherapy for IPF and NSCLC. Given that treatment with nintedanib has a higher risk of causing ischemic heart disease,^7^ we hypothesized that pirfenidone might be a safer alternative for reducing chemotherapy‐associated AE‐IPF in patients with IPF and NSCLC.

The present study therefore aimed to assess the safety and effectiveness of pirfenidone combined with carboplatin‐based chemotherapy or ICIs for patients with IPF and NSCLC.

## Methods

### Study patients and inclusion criteria

**Figure 1:**
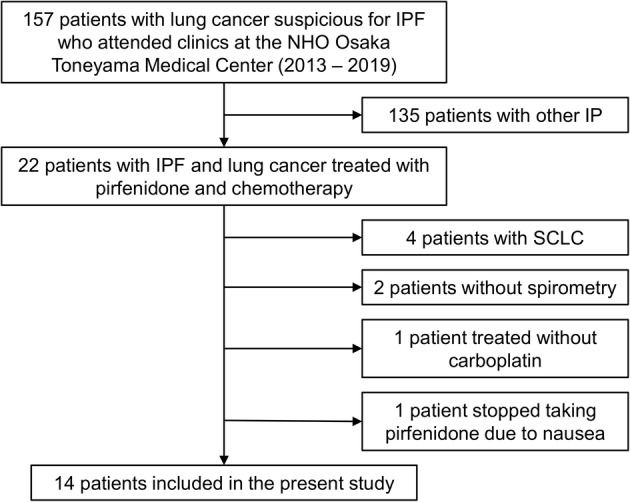
Patient inclusion flowchart. IP, interstitial pneumonia; IPF, idiopathic pulmonary fibrosis; NHO, National Hospital Organization; SCLC, small cell lung cancer.

A total of 157 Japanese patients with interstitial pneumonia and lung cancer receiving treatment at the National Hospital Organization Osaka Toneyama Medical Center between January 2013 and December 2019 were screened. A patient inclusion flowchart is presented in Fig 1. Those patients who had secondary interstitial pneumonia were excluded. Usual interstitial pneumonia (UIP) was diagnosed based on the presence of a UIP pattern on high‐resolution computed tomography (CT) without surgical lung biopsy or specific combinations of high‐resolution CT findings and surgical lung biopsy patterns.^24^ Patients with small cell lung cancer, as well as those who did not undergo spirometry within one month after initiating chemotherapy, were excluded. Patients who had inoperable NSCLC due to recurrence or intolerability and/or disease progression were included. Moreover, only patients who started carboplatin‐based regimens as first‐line chemotherapy at least three days after the prescription of pirfenidone were included. One patient who discontinued pirfenidone before the initiation of chemotherapy for NSCLC was excluded. In total, 14 patients qualified for this study and were evaluated using examinations and analysis described in the following sections.

### Study design

Medical records of patients were retrospectively reviewed. All patients were evaluated to determine the NSCLC stage before treatment initiation or NSCLC progression through complete medical histories and physical examinations, chest radiography, chest and abdomen CT, and other staging procedures, including brain magnetic resonance imaging. All cancer lesions were measurable using the Response Evaluation Criteria in Solid Tumors guideline.^25^ Patients were treated with pirfenidone combined with carboplatin and weekly nab‐paclitaxel or S‐1 as first‐line chemotherapy. After confirming NSCLC progression, patients received other cytotoxic agents or ICIs. Regardless of lung cancer progression or changes in chemotherapy, patients continued to receive pirfenidone for as long as possible. When pirfenidone was switched to nintedanib during late‐line chemotherapy, patient tracking was discontinued. Progression‐free survival (PFS) for lung cancer and IPF, OS, and chemotherapy‐associated AE‐IPF were calculated. The cutoff date for data collection was 31 December 2019. The primary endpoint of this study was AE‐IPF associated with first‐line chemotherapy using pirfenidone and carboplatin. The secondary endpoints included ICIs‐associated AE‐IPF and cumulative incidence of AE‐IPF within one year and until death. The Institutional Review Board of the National Hospital Organization Osaka Toneyama Medical Center approved the study protocols and chose an opt‐out system for obtaining patients' informed consent (approval number: TNH‐P‐2020016).

### Spirometry

Spirometry was performed within one month before pirfenidone prescription. All patients underwent spirometry using the CHESTAC 8800 spirometer (Chest M.I., Inc., Tokyo, Japan) according to the recommendations of the American Thoracic Society (ATS) and the European Respiratory Society (ERS).^26^ Short‐acting β_2_‐agonists were not used for at least 12 hours before tests in all patients. Long‐acting β_2_‐agonists and long‐acting antimuscarinic agents were not withdrawn before spirometry. Predicted FVC and forced expiratory volume in one second (FEV_1_) were calculated according to the formula for Japanese patients developed by the Japanese Respiratory Society.^27^

### **Carbon monoxide diffusing capacity** **(DL**_**CO**_**)**

DL_CO_ was measured using the CHESTAC 8800 spirometer (Chest M.I., Inc., Tokyo, Japan) and a single‐breathing method according to the recommendations of the ERS and ATS standard criteria.^28^ DL_CO_ values were adjusted using hemoglobin levels when possible.

### GAP and mGAP indices

GAP and mGAP indices were calculated for all patients included herein.^4^, ^6^ The GAP index incorporated gender, age, FVC % predicted, and DL_CO_ % predicted.^4^ The final GAP score ranged from 0 to 8, and it was used to classify patients as stage I (0 to 3 points, mild disease), stage II (4 to 5 points, moderate disease), or stage III (6 to 8 points, severe disease). The mGAP index incorporated all variables of the GAP index except for DL_CO_. The final mGAP score ranged from 0 to 5 and was used to classify patients as stage I (0 to 3 points, mild disease) or stage II (4 to 5 points, severe disease).^6^

### Treatment regimens

Patients initially received pirfenidone, and first‐line chemotherapy was initiated at least three days after the pirfenidone prescription. Pirfenidone was increased from 600 mg daily to a maximum of 1200 mg daily. As first‐line chemotherapy for NSCLC, patients received carboplatin (area under the curve = 6, day 1) and weekly nab‐paclitaxel (100 mg/m^2^, days 1, 8, and 15) or S‐1 (80–120 mg daily, days 1–14). Each treatment cycle was repeated four to six times unless there was evidence of NSCLC progression or unacceptable toxicity was confirmed, or the patient/physician decided to discontinue treatment. Subsequent doses were modified by the physician based on hematological and nonhematological toxicities. After discontinuing first‐line chemotherapy, patients received nab‐paclitaxel (100 mg/m^2^, days 1, 8, and 15), S‐1 (80–120 mg daily, days 1–14), vinorelbine (25 mg/m^2^, days 1 and 8), nivolumab (240 mg daily, day 1) or pembrolizumab (200 mg daily, day 1). Pirfenidone was continued as long as possible regardless of chemotherapy changes or NSCLC progression. In one patient, pirfenidone was switched to nintedanib at the physician's discretion without IPF progression and AE‐IPF. All patients had adequate organ function before treatment initiation. Peripheral blood and biochemistry examinations were repeated at least once per cycle.

### Statistical analysis

PFS for lung cancer was defined as the time from the initial administration of first‐line chemotherapy until the date of confirmed disease progression or cancer‐induced death. OS was defined as the time from the initial administration of first‐line chemotherapy until death. PFS for IPF was defined as the time from the administration of first‐line chemotherapy until the date of confirmed IPF progression. IPF progression was defined as ≥10% decline in percentage predicted FVC, ≥15% decline in percentage predicted DL_CO_, or IPF‐induced death. AE‐IPF was defined as an acute, clinically significant respiratory deterioration characterized by evidence of new widespread alveolar abnormality according to the ATS recommendation.^29^ Chemotherapy‐associated AE‐IPF was defined as AE‐IPF that occurred within 30 days from the last chemotherapy administration and/or until the initiation of next‐line chemotherapy.

The median probabilities for OS and PFS for lung cancer and IPF were calculated using the Kaplan–Meier method. Chemotherapy‐associated AE‐IPF was calculated regarding all treatment regimens. In addition, the cumulative incidences of AE‐IPF within one year from first‐line chemotherapy administration and over the entire observational period were calculated using Gray's test. For all analyses, *P*‐values < 0.05 were considered statistically significant. All statistical analyses were performed using EZR Version 1.38 (based on R Version 3.5.2 and R commander Version 2.5–1; Jichi Medical University Saitama Medical Center, Saitama, Japan).^30^

## Results

### Baseline characteristics

Among the 14 patients included in the present study, 10 were diagnosed with CPFE (Table 1). Most patients were classified as GAP stage I, although two patients could not be evaluated using the GAP index due to missing DL_CO_ data. Moreover, 12 and two patients were classified as mGAP stage I and II, respectively. Oral corticosteroids were administered in six patients in addition to pirfenidone. All patients were diagnosed with NSCLC using transbronchial biopsy and exhibited Eastern Cooperative Oncology Group Performance Status (PS) of 0 or 1. Histology revealed squamous cell carcinoma (five patients) and adenocarcinoma (four patients). Either carboplatin and nab‐paclitaxel (eight patients), or S‐1 (six patients) were used as first‐line chemotherapy for NSCLC (Table 2). Although all patients tolerated first‐line chemotherapy, two second‐line chemotherapy regimens (carboplatin and S‐1 and S‐1 monotherapy) were discontinued due to skin rashes. Moreover, carboplatin and nab‐paclitaxel as second‐line chemotherapy and nab‐paclitaxel monotherapy as third‐line chemotherapy were discontinued due to renal dysfunction and increased susceptibility to infection, respectively. All treatment regimens, except for the four mentioned, were continued until NSCLC progression, or treatment cessation due to worsened PS or AE‐IPF. ICIs were used as second‐ or third‐line chemotherapy in four patients (one patient received nivolumab and three patients received pembrolizumab; Table 2). All four patients were classified as GAP and mGAP stage I, showing mild IPF (Table 3). Throughout the entire chemotherapy period, all patients tolerated the combination of pirfenidone and cytotoxic agents or ICIs. Although pirfenidone was switched to nintedanib in one patient at the physician's discretion after the end of third‐line chemotherapy, all other patients continued pirfenidone over the entire chemotherapy period.

**Table 1:** Baseline characteristics (*n* = 14)

Characteristics		
Age, years	69.6 ± 5.9	61–79
Sex, male/female, *n*	12/2	
Height, cm	165.1 ± 7.6	152.4–179.2
Weight, kg	60.3 ± 8.2	50.3–76.6
BMI, kg m^−2^	22.1 ± 2.8	17.7–27.1
Smoking, pack‐years	44 ± 13	16–60
mMRC dyspnea scale, *n*		
0/1/2/3/4	1/9/4/0/0	
LDH, U/L	248 ± 117	125–617
KL‐6, U/mL	1108 ± 1180	414–4971
Medications		
Pirfenidone, *n* (%)	14 (100.0)	
Oral corticosteroids, *n* (%)	6 (42.9)	
Nintedanib, *n* (%)	1 (7.1)	
Spirometry		
FEV_1_, L	2.16 ± 0.53	1.20–2.83
FEV_1_, % predicted	80.2 ± 18.5	38.1–111.5
FEV_1_/FVC, %	74.0 ± 7.4	61.5–84.9
FVC, L	2.93 ± 0.77	1.95–4.30
FVC, % predicted	87.5 ± 20.9	51.2–131.3
DL_CO_, mL/minute/mmHg	11.04 ± 2.92	5.58–16.76
DL_CO_, % predicted	68.3 ± 21.1	33.2–99.9
GAP index	3.3 ± 1.2	2–7
GAP stage, *n*		
I/II/III/not applicable	10/1/1/2	
mGAP index	2.9 ± 0.7	2–4
mGAP stage, *n*		
I/II	12/2	
CPFE, *n* (%)	10 (71.4)	
ECOG performance status, *n*		
0/1/2/3/4	1/13/0/0/0	
Histology of lung cancer, *n* (%)		
Adenocarcinoma	4 (28.6)	
Squamous cell carcinoma	5 (35.7)	
NSCLC, NOS	5 (35.7)	
EGFR mutation/ALK fusion gene, *n* (%)		
Wild‐type	14 (100.0)	
Clinical stage, *n*		
IIIA/IIIB/IVA/IVB/recurrent	5/5/2/1/1	

Data are presented as mean ± SD and minimum and maximum values, unless otherwise stated.

ALK, anaplastic lymphoma kinase; BMI, body mass index; CPFE, combined pulmonary fibrosis and emphysema; DL_CO_, carbon monoxide diffusing capacity; ECOG, Eastern Cooperative Oncology Group; EGFR, epidermal growth factor receptor; FEV_1_, forced expiratory volume in one second; FVC, forced vital capacity; GAP, gender, age, and physiology; LDH, lactate dehydrogenase; mGAP, modified GAP; mMRC, modified Medical Research Council; NOS, not otherwise stated; NSCLC, non‐small cell lung cancer; SD, standard deviation.

**Table 2:** Chemotherapy regimens for patients with non‐small cell lung cancer (*n* = 14)

Patient	First	Second	Third	Fourth	AE‐IPF
1	CBDCA + nab‐PTX	S‐1			−
2	CBDCA + nab‐PTX	CBDCA + S‐1†	VNR	S‐1 (rechallenge)‡	+
3	CBDCA + nab‐PTX				+
4	CBDCA + nab‐PTX				−
5	CBDCA + nab‐PTX	S‐1	Pembrolizumab		−
6	CBDCA + nab‐PTX				−
7	CBDCA + nab‐PTX	VNR	S‐1		−
8	CBDCA + nab‐PTX	S‐1†	VNR‡		+
9	CBDCA + S‐1	CBDCA + nab‐PTX†	S‐1 (rechallenge)		−
10	CBDCA + S‐1	CBDCA + nab‐PTX			+
11	CBDCA + S‐1	CBDCA + nab‐PTX			−
12	CBDCA + S‐1	Pembrolizumab	nab‐PTX†		−
13	CBDCA + S‐1	Nivolumab	CBDCA + nab‐PTX		−
14	CBDCA + S‐1	nab‐PTX	Pembrolizumab		−

† Treatment regimens discontinued due to adverse reactions other than AE‐IPF.

‡ Treatment regimens induced chemotherapy‐associated AE‐IPF.

+ and −, Presence and absence of AE‐IPF until death, respectively.

CBDCA, carboplatin; AE‐IPF, acute exacerbation of idiopathic pulmonary fibrosis; nab‐PTX, nanoparticle albumin‐bound PTX; PTX, paclitaxel; VNR, vinorelbine.

**Table 3:** Characteristics of patients receiving immune checkpoint inhibitors (*n* = 4)

Patient	Age	Histology	ICIs	FVC, L	FVC, % predicted	DL_CO_, mL/min/mmHg	DL_CO_, % predicted	GAP stage	mGAP stage
1	61	SCC	Pembrolizumab	2.75	71.8	11.27	61.0	I	I
2	78	SCC	Pembrolizumab	2.40	81.1	10.91	87.8	I	I
3	69	NSCLC	Pembrolizumab	1.96	84.8	8.29	53.9	I	I
4	73	NSCLC	Nivolumab	4.30	118.5	16.76	99.9	I	I

DL_CO_, carbon monoxide diffusing capacity; FVC, forced vital capacity; GAP, gender, age, and physiology; ICIs, immune checkpoint inhibitors; mGAP, modified GAP; NSCLC, non‐small cell carcinoma; SCC, squamous cell carcinoma.

### Survival analysis for IPF and lung cancer progression

**Figure 2:**
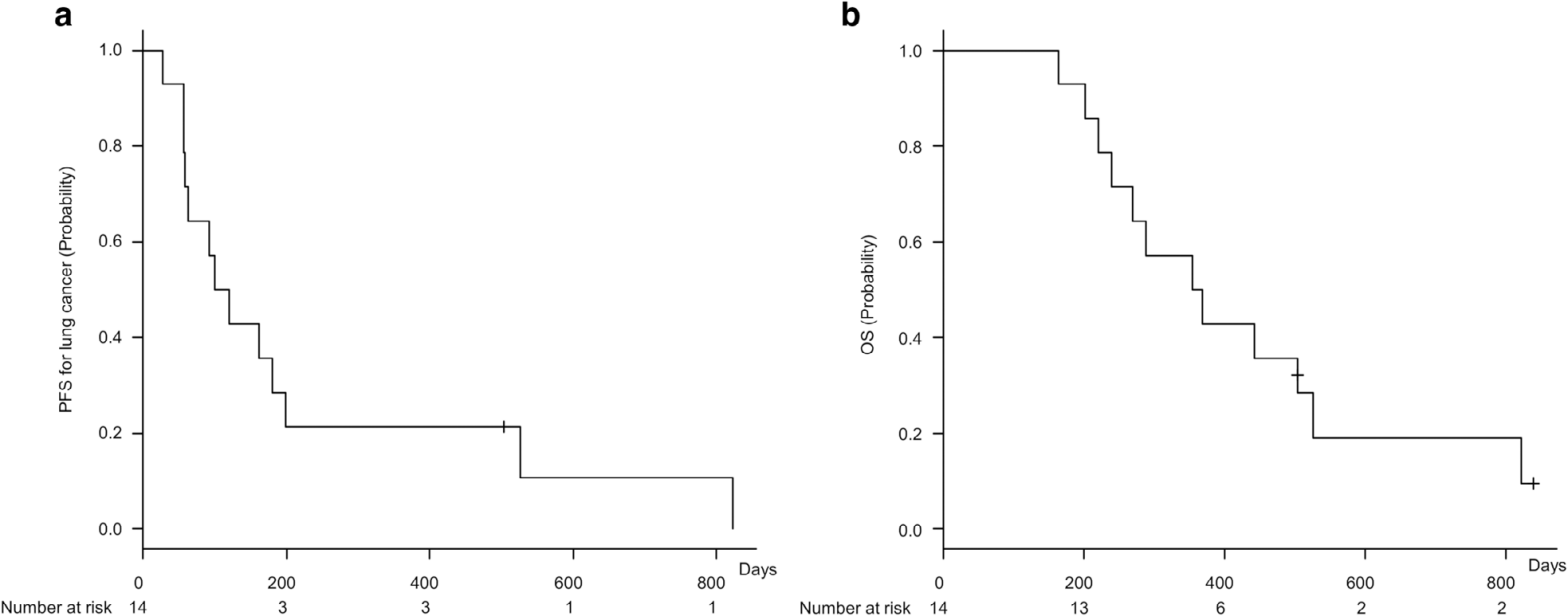
Progression‐free survival (PFS) for lung cancer and overall survival (OS) in patients with idiopathic pulmonary fibrosis and non‐small cell lung cancer (*n* = 14). (**a**) Kaplan–Meier curve of PFS for lung cancer. Median PFS for lung cancer was 110 days (95% confidence interval [CI]: 57–199 days). (**b**) Kaplan–Meier curve of OS. Median OS was 362 days (95% CI: 220–526 days).

**Figure 3:**
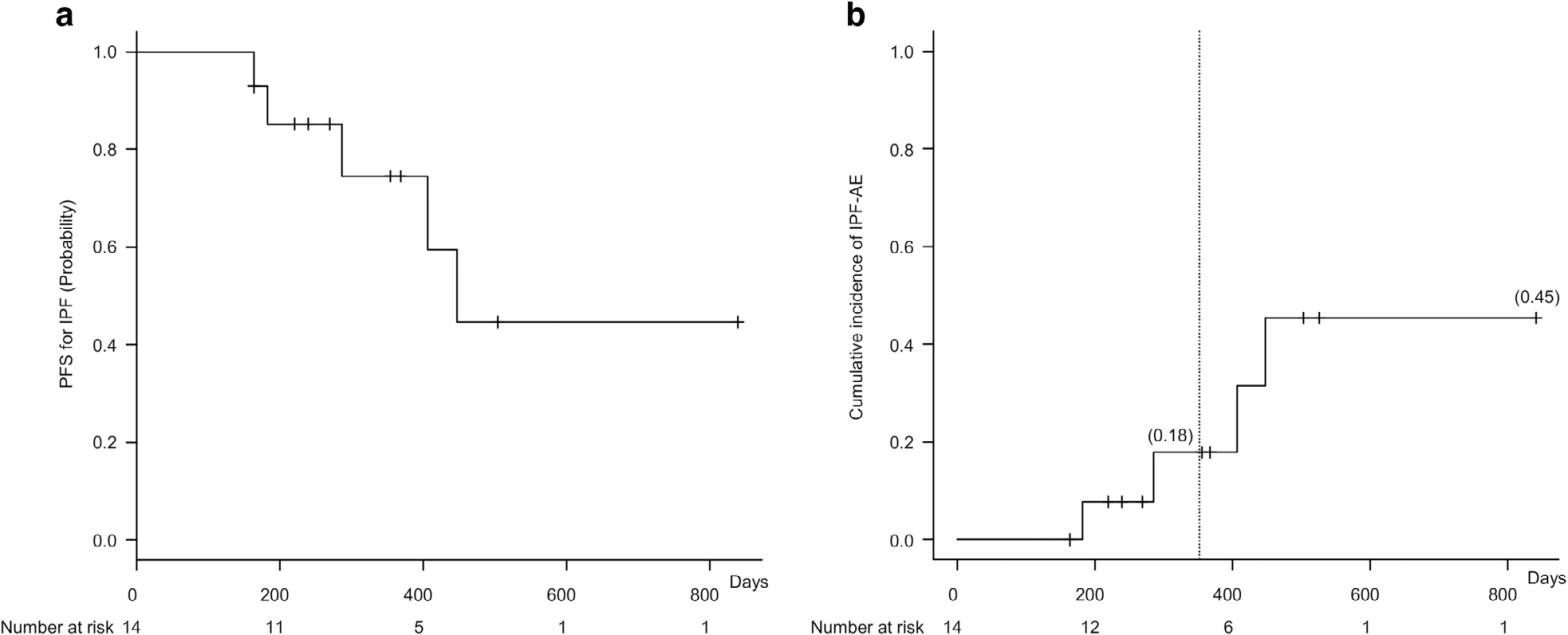
Progression‐free survival (PFS) for idiopathic pulmonary fibrosis (IPF) and cumulative incidence of acute exacerbation of IPF (AE‐IPF) (*n* = 14). (**a**) Kaplan–Meier curve of PFS for IPF. PFS for IPF was 447 days (95% CI: 286–indeterminate days). (**b**) Cumulative incidence of AE‐IPF within one year from the initiation of first‐line chemotherapy and throughout the entire period were 18% and 45%, respectively.

Median PFS for lung cancer was 110 days (95% confidence interval [CI]: 57–199 days), and median OS was 362 days (95% CI: 220–526 days; Fig 2). Causes of death included NSCLC progression (10 patients) and AE‐IPF (four patients). Notably, none of the patients developed AE‐IPF associated with first‐line chemotherapy (Table 4). Moreover, none of the patients receiving ICIs experienced AE‐IPF throughout the entire observational period. However, among the four patients who did experience AE‐IPF, two received S‐1 as fourth‐line chemotherapy or vinorelbine as third‐line chemotherapy. PFS for IPF was 447 days (95% CI: 286–indeterminate days). The cumulative incidence of AE‐IPF within one year and throughout the entire period was 18% and 45%, respectively (Fig 3).

**Table 4:** Occurrence of acute exacerbations of idiopathic pulmonary fibrosis (AE‐IPF) (*n* = 14)

Variable	Events
Until the initiation of second‐line chemotherapy, *n* (%)	0 (0.0)
Within 30 days from the last first‐line chemotherapy administration, *n* (%)	0 (0.0)
Entire observation period, *n* (%)	4 (28.6)

## Discussion

The results of the present study showed two major findings regarding the utility of pirfenidone: (i) pirfenidone combined with carboplatin‐based chemotherapy was a safe and effective first‐line chemotherapy with low incidence of AE‐IPF for patients with IPF and NSCLC, particularly those with good PS and mGAP stage I; and (ii) pirfenidone combined with ICIs was safe for patients with IPF and NSCLC. To the best of our knowledge, this is the first study which has assessed the safety and effectiveness of pirfenidone in combination with cytotoxic agents or ICIs for patients with IPF and NSCLC.

Pirfenidone inhibits transforming growth factor (TGF)‐β and suppresses epithelial‐mesenchymal transition (EMT).^31^, ^32^ EMT is a fundamental process in which epithelial cells lose their polarity and transform into mesenchymal cells, the subtypes of which are associated with organ fibrosis and neoplastic environment.^33^ Type 1 EMT is associated with implantation and embryonic gastrulation, while type 2 EMT involves the transformation of epithelial cells into mesenchymal cells, which finally induces fibroblasts in the context of inflammation and leads to organ fibrosis.^33^ Meanwhile, type 3 EMT occurs in neoplastic cells and allows primary epithelial cancer cells to invade adjacent organs, enter the circulation, and metastasize to distant organs.^33^ Pirfenidone reportedly inhibited type 2 and 3 EMT and suppressed organ fibrosis and tumor progression in vitro and in vivo.^31^, ^34^ Apart from inhibiting EMT, an earlier study reported that pirfenidone inhibited TGF‐β and induced cell cycle arrest in NSCLC cells,^35^ suggesting its ability to inhibit tumor progression, invasion, and metastasis by inhibiting multiple TGF‐β‐associated pathways in NSCLC. In fact, a retrospective observational study showed that patients with IPF prescribed pirfenidone had a lower incidence of lung cancer.^36^ Given these earlier studies, pirfenidone exhibits antifibrotic effects and might potentially exert antitumor effects in patients with IPF and NSCLC.

The present study showed that pirfenidone combined with carboplatin‐based chemotherapy might be a safe and effective first‐line chemotherapy for patients with IPF and NSCLC given that the combination did not induce AE‐IPF in any of the patients (Table 4). Moreover, the cumulative incidence of AE‐IPF within one year was 18% in this study (Fig 3), which was lower than that presented in an earlier report investigating patients with IPF and NSCLC who did not receive pirfenidone (30% in mGAP stage I and 82% in mGAP stage II).^6^ These results therefore suggest that pirfenidone might have a prophylactic effect against chemotherapy‐associated AE‐IPF in patients with IPF and NSCLC. Since the OS in this study (11.9 months) was relatively longer than that for patients with mGAP stage I in the earlier study (10.3 months),^6^ pirfenidone might potentially have prolonged OS by reducing AE‐IPF. However, given that late‐line chemotherapy comprising S‐1 and vinorelbine induced AE‐IPF, the combination of pirfenidone might be safe only for patients with good PS and mGAP stage I. Moreover, 14 of 15 patients who could tolerate pirfenidone continued its use till the last late‐line chemotherapy administration. This suggests that patients with IPF and NSCLC can tolerate the combination of pirfenidone and carboplatin‐based chemotherapy. Therefore, further prospective studies assessing the safety and effectiveness of pirfenidone are warranted to validate the results in a larger cohort.

Pirfenidone combined with ICIs, particularly anti‐PD‐1 antibodies, might be safe for patients with IPF and NSCLC. ICIs have been developed to target immune checkpoints commonly used by cancer cells in immune editing and block cancer cell evasion from immune detection.^37^ By blocking PD‐1, ICIs invigorate T lymphocytes and enable CD8^+^ T lymphocytes to engage in the cytotoxic killing of cancer cells.^37^ Meanwhile, fibroblasts and CD4^+^ T lymphocytes in IPF have been shown to express PD‐L1 and PD‐1, respectively;^38^, ^39^ the upregulation of both is associated with pulmonary fibrosis.^38^, ^39^ Hence, PD‐1/PD‐L1 checkpoint might be associated with fibroblast immune editing and subsequent invasion and metastases to other organs in IPF. In addition, PD‐1/PD‐L1 checkpoint inhibitors are related to TGF‐β production and pulmonary fibrosis, which is independent of immune regulation.^37^ Therefore, ICIs can be considered safe for patients with IPF,^40^ while the combination of pirfenidone and ICIs may be used without IPF progression because the combination variably blocks TGF‐β and the crosstalk between CD4^+^ T lymphocytes and fibroblasts through the PD‐1/PD‐L1 checkpoint. In fact, the results of this study showed that pirfenidone combined with nivolumab or pembrolizumab was safe in four patients with IPF and NSCLC (Table 2). Therefore, further studies for assessing the safety and effectiveness of combined chemotherapy using antifibrotic agents and ICIs are needed.

The present study has some limitations. First, this was a retrospective single‐center study, and selection bias might have affected the findings. Second, this study did not include controls who were not prescribed pirfenidone due to the small number of patients. Thus, further studies are warranted to validate the results. Third, the present study mostly included patients with mGAP stage I and PS of 0 or 1. Therefore, the safety of pirfenidone combined with carboplatin‐based chemotherapy should be further assessed in patients with mGAP stage II and/or PS 2. Fourth, this study did not include patients with epithelial growth factor receptor mutation or anaplastic lymphoma kinase fusion gene, and studies which expand the present results in these populations are necessary. Finally, given that this study included only a small number of patients with CPFE and IPF without emphysema, further studies investigating the prophylactic effect of pirfenidone against AE‐IPF are needed.

In conclusion, the present study evaluated the safety and effectiveness of pirfenidone combined with carboplatin‐based chemotherapy or ICIs in patients with IPF and NSCLC. Pirfenidone combined with carboplatin‐based chemotherapy, particularly with nab‐paclitaxel or S‐1, might be safe and effective in IPF and NSCLC patients with good PS and mGAP stage I. Moreover, this study showed that pirfenidone combined with ICIs may be used safely as late‐line chemotherapy in patients with IPF and NSCLC. Although this pilot study was too small to conclude the safety and effectiveness of pirfenidone combined with cytotoxic agents or ICIs, our findings provide a foundation for further prospective investigations regarding pirfenidone, cytotoxic agents, and ICIs in patients with IPF and NSCLC.

## Disclosure

The authors declare no potential conflicts of interest.
